# Tumor Necrosis Factor Alpha Signaling and Organogenesis

**DOI:** 10.3389/fcell.2021.727075

**Published:** 2021-07-30

**Authors:** Kai You, Hui Gu, Zhengwei Yuan, Xuewen Xu

**Affiliations:** ^1^Department of Pediatrics, Shengjing Hospital of China Medical University, Shenyang, China; ^2^Key Laboratory of Health Ministry for Congenital Malformation, Shengjing Hospital of China Medical University, Shenyang, China; ^3^Department of Urology, Shengjing Hospital of China Medical University, Shenyang, China

**Keywords:** tumor necrosis factor alpha, cell communication, development, morphogenesis, organogenesis

## Abstract

Tumor necrosis factor alpha (TNF-α) plays important roles in processes such as immunomodulation, fever, inflammatory response, inhibition of tumor formation, and inhibition of viral replication. TNF-α and its receptors are ubiquitously expressed in developing organs and they regulate the survival, proliferation, and apoptosis of embryonic stem cells (ESCs) and progenitor cells. TNF-α is an important inflammatory factor that also regulates the inflammatory response during organogenesis, and its cytotoxic effects can interfere with normal developmental processes, even leading to the onset of diseases. This review summarizes the various roles of TNF-α in organogenesis in terms of its secreting pattern, concentration-dependent activities, and interactions with other signaling pathways. We also explored new potential functions of TNF-α.

## Introduction

Tumor necrosis factor alpha (TNF-α) belongs to the TNF superfamily (Aggarwal, 2003) of proteins with highly similar structures and conserved interaction profiles (Bodmer et al., 2002). A macrophage cytotoxic factor was originally discovered during 1975 that could kill mouse fibrosarcoma L-929 cells and was thus termed “tumor necrosis factor” (Carswell et al., 1975). TNF-α plays important roles in various biological processes, such as immunomodulation, fever, inflammatory response, inhibition of tumor formation, and inhibition of virus replication (Bradley, 2019). TNF-α is encoded by a 3-kb gene located on chromosome 6p21.3 and it comprises four exons (Old, 1985). The precursor of TNF-α (pro-TNF-α) is a type II transmembrane protein with a molecular weight of 26 kDa, consisting of mature TNF-α and a leader sequence, which contains a cytoplasmic domain, a transmembrane domain, and an extracellular domain. Synthesized pro-TNF is incorporated into the cell membrane and rapidly forms a homotrimer, which is then proteolytically cleaved by a multidomain metalloproteinase called TNF-α converting enzyme to release 17-kDa soluble (mature) TNF-α (Tang et al., 1996). Despite being a precursor, pro-TNF also exhibits biological activities; for instance, the homolog of pro-TNF, Eiger, induces apoptosis in compound eyes of Drosophila by activating the c-Jun N-terminal kinases (JNKs) signaling pathway (Igaki et al., 2002), and concentric cardiac hypertrophy occurs in transgenic mice with up-regulated pro-TNF expression (Dibbs et al., 2003). The binding of TNF-α to its receptors activates three types of intracellular signaling pathways, including the NF-κ B-, MAPK- JNK-, and caspase-8-mediated pathways, to promote various biological functions, such as the inflammatory response, as well as cell survival, proliferation, differentiation, and apoptosis (Adrain et al., 2012). The physiological roles of TNF-α in developmental processes have recently gained attention. TNF-α promotes the growth of intestinal epithelium in fetuses by stimulating the development of intestinal stem cells (Schreurs et al., 2019). TNF-α also promotes the apoptosis of cardiac valve interstitial cells (VICs). TNF-α-knockout mice developed VIC hypertrophy at 16 days post-partum, indicating that TNF-α plays an important role in the development of cardiac valves (Wang et al., 2017).

This review summarizes the roles of TNF-α in the development of various organs and associated diseases. We also highlight the mechanisms of TNF-α from multiple aspects, including its effects on stem cells/progenitor cells, secreted forms, concentration-dependent activities, and interactions with other signaling pathways.

## Roles, Actions, and Effects of TNF-α

### TNF-α Participates in the Regulation of Organogenesis

**FIGURE 1:**
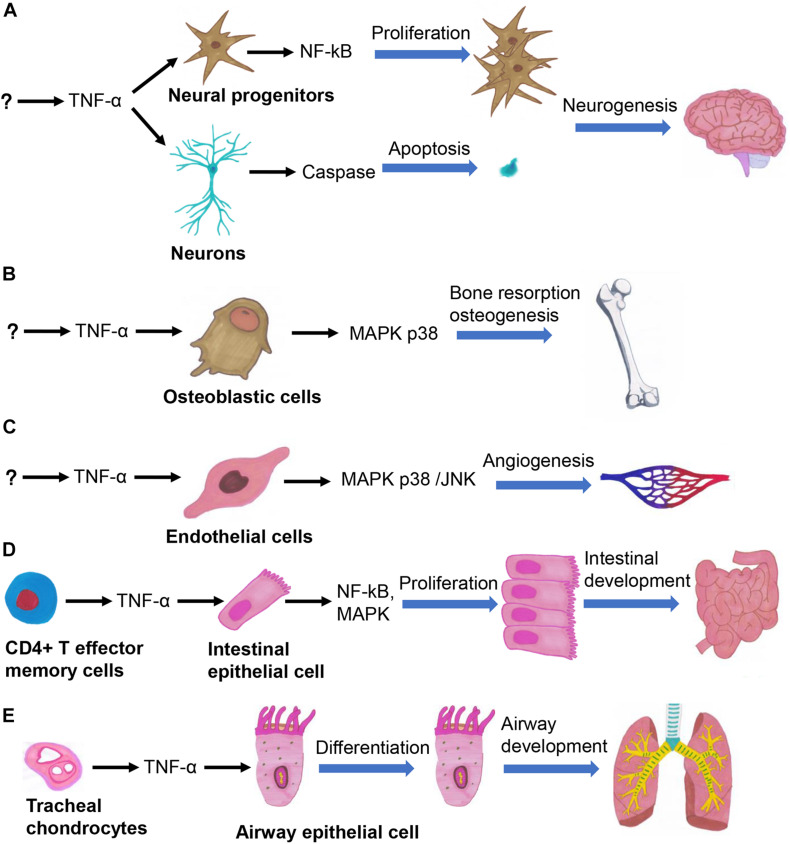
TNF-α regulates several important biological processes in organogenesis, such as the neurogenesis **(A)**, osteogenesis **(B)**, angiogenesis **(C)**, intestinal development **(D)**, and airway development **(E)**.

The effects of TNF-α can be traced back to gastrulation, during which it promotes embryonic differentiation and cell apoptosis (Sanders et al., 1997). Organogenesis is precisely regulated by a complex signaling network (Durdu et al., 2014), in which TNF-α is involved in regulating the development of multiple organs (Figure 1). TNF-α plays a central role in the process of neurogenesis in embryos and neonates by regulating the survival, proliferation, and differentiation of neural progenitor cells (NPCs) (Bernardino et al., 2008; Lan et al., 2012). TNF-α participates in various stages of brain development, by increasing the numbers of neurons in the early stage of embryonic development through activating the NF-κB signaling pathway, and induces neuronal apoptosis in the late stage of embryonic development by activating the caspase pathway (Figure 1A; Doherty, 2007). TNF-α promotes the differentiation of keratinocytes in neonates by increasing the rate of cornified envelope formation. It also promotes progression of the hair follicle cycle from the growth (anagen) to the regression (catagen) phase, thus playing important regulatory roles in epidermal development and hair follicle morphogenesis (Pillai et al., 1989; Tong and Coulombe, 2006). TNF-α promotes the proliferation of bone marrow-derived mesenchymal stem cells (BMSCs), osteoclast progenitor cells, and chondrocytes (Enomoto et al., 1990; Van Der Pluijm et al., 1991; Fang et al., 2019). TNF-α also promotes the migration of BMSCs without relying on the NF-κB signaling pathway (Sullivan et al., 2014). TNF-α activates the p38 MAPK signaling pathway in osteoblasts and chondrocytes to enhance bone resorption, thus promoting bone growth (Figure 1B; Tashjian et al., 1987; Kumar et al., 2001). TNF-α seemingly exhibits various effects across different developmental stages; for instance, it inhibits (Gilbert et al., 2000) and promotes (Sidney et al., 2014) the differentiation of osteoblasts in fetal and neonatal rats, respectively. TNF-α secreted by valvular endothelial cells in embryonic mice induces the apoptosis of VICs, whereby TNF-α-knockout mice have thickened heart valves (Wang et al., 2017). Chick embryo chorioallantoic membrane assays have shown that TNF-α promotes angiogenesis (Figure 1C; Olivo et al., 1992; Fang et al., 2019). The embryos of TNF-α-knockout mice are more prone to developing limb deformities after exposure to cyclophosphamide, confirming that TNF-α functions as a cytokine that protects embryos against teratogens (Torchinsky et al., 2003). TNF-α promotes the growth of intestinal stem cells in the human fetus (Figure 1D; Schreurs et al., 2019). The onset of severe hepatic dysplasia in embryos of TNF-α-knockout zebrafish showed that TNF-α plays an important role in liver development (Qi et al., 2010). TNF-α secreted by tracheal cartilage regulates the differentiation of airway epithelial cells in embryos (Figure 1E; Turcatel et al., 2017). Besides, mechanical ventilation can lead to bronchopulmonary dysplasia in TNF-α-knockout mice by inducing the transforming growth factor (TGF) signaling pathway, indicating that the balance between TNF-α and TGF signaling is essential for airway development (Ehrhardt et al., 2016). Therefore, TNF-α participates in the regulation of cell survival and proliferation by activation of NF-κB signaling, cell differentiation and proliferation by activation of MAPK signaling, and apoptosis by activation of caspase-8 signaling, so that it plays important roles in the development of various organs. Further studies are required to gain insights into the regulatory mechanism of TNF-α in organ development and cellular signaling pathways.

### Effects of TNF-α on Stem Cells/Progenitors

**FIGURE 2:**
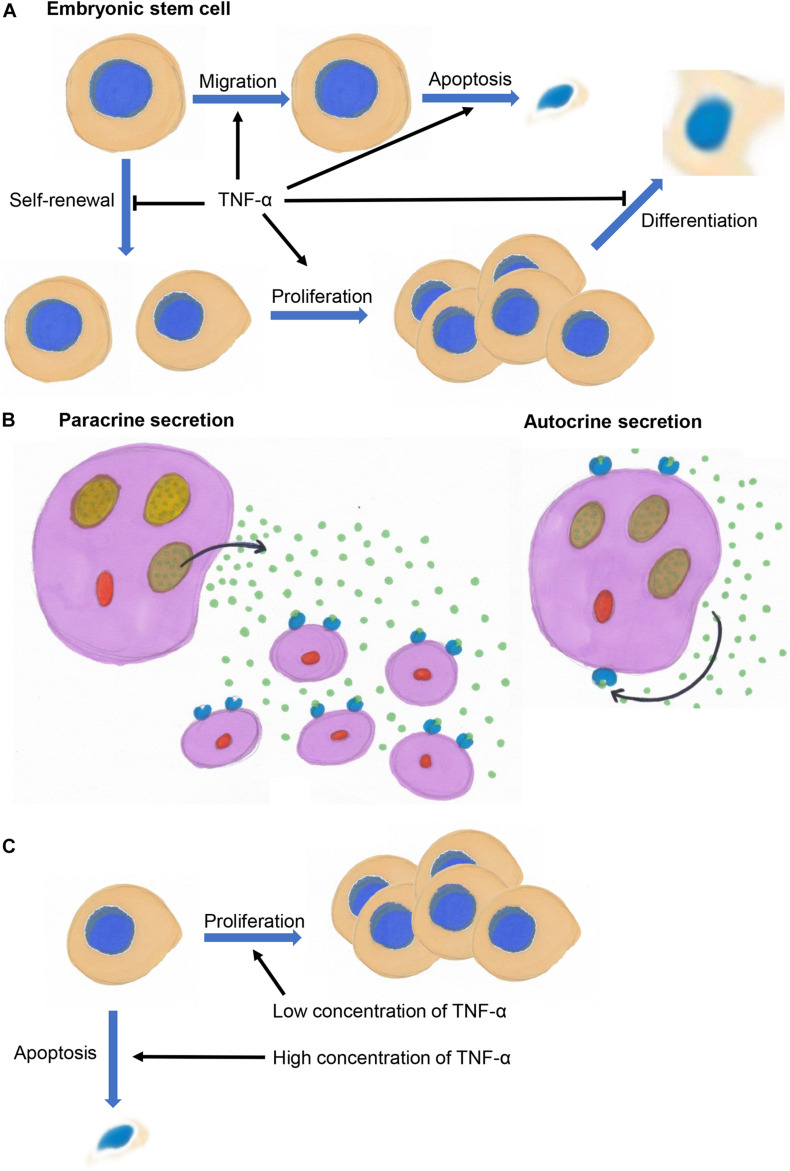
**(A)** TNF-α inhibits the self-renewal of embryonic stem cells and promotes their migration, and inhibits the differentiation of progenitor cells and promotes their proliferation and survival. **(B)** TNF-α acts on its secreting cells (autocrine signaling) or surrounding cells (paracrine signaling). **(C)** TNF-α promotes cell proliferation at low concentrations and inhibit cell proliferation and induce apoptosis at high concentrations.

Tumor necrosis factor alpha might exert different effects on embryonic stem cells (ESCs), progenitor cells, and differentiated cells. It not only inhibits the self-renewal of mouse ESCs but also induces their apoptosis and inhibits their differentiation into embryos (Figure 2A; Wuu et al., 1998). TNF-α promotes the migration of ESCs by binding to TNF receptor 2 to activate p38 and JNKs *in vivo* and *in vitro* (Chen et al., 2003).

Tumor necrosis factor alpha is also involved in regulating the proliferation, apoptosis, and differentiation of progenitor cells. It promotes the proliferation and differentiation of neuroblasts into astrocytes in the human fetal cortex (Peng et al., 2008; Lan et al., 2012), inhibits the differentiation of cortical oligodendrocyte precursor cells and induces their apoptosis in neonatal rats (Su et al., 2011; Bernardo et al., 2017). TNF-α promotes the differentiation of NPCs in the subventricular zone of neonatal mice. Moreover, low and high concentrations of TNF-α, respectively, promote the proliferation and apoptosis of NPCs (Bernardino et al., 2008). TNF-α promotes the survival of human embryonic NPCs by activating NF-κB signaling pathway (Kim et al., 2018). TNF-α inhibits the proliferation of hippocampal precursor cells in mice (Wang et al., 2018) and the differentiation of NPCs into neurons in embryos (Liu et al., 2005), and promotes the proliferation of osteoclast progenitor cells in embryonic mice (Van Der Pluijm et al., 1991). TNF-α plays an important regulatory role in the differentiation of fetal thymic and lymphoid precursor cells (Zúñiga-Pflücker et al., 1995). TNF-α also inhibits the differentiation of Schwann cells in neonatal rats, osteoblasts in fetal rats, and colonic epithelial cells in human fetuses (Gilbert et al., 2000, 2002; Lisak et al., 2001; Hýžïalová et al., 2008), and induces the apoptosis of oocytes in neonatal rats (Morrison and Marcinkiewicz, 2002). Therefore, TNF-α tends to promote proliferation and inhibit differentiation of the progenitors by activating NF-κB signaling, while with regards to stem cells and differentiated cells, TNF-α tends to induce their apoptosis.

### Secreted Forms and Biological Functions of TNF-α

Although transmembrane TNF-α (pro-TNF) is biologically active, TNF-α primarily exerts autocrine and paracrine functions in a soluble, trimeric form during developmental processes. TNF-α acts as an autocrine and paracrine growth factor that stimulates the proliferation of hematopoietic cells and B cells (Figure 2B; Boussiotis et al., 1994). Autocrine TNF-α signaling is required for macrophage maturation (Witsell and Schook, 1992; Boyle et al., 2003; Chen et al., 2004). Autocrine TNF-α signaling promotes the survival and differentiation of monocytes into dendritic cells (Lehner et al., 2012). Autocrine TNF-α signaling is also involved in the regulation of growth, differentiation, and maturation of lymphokine-activated killer T cells (Innins et al., 1992). TNF-α regulates the differentiation of osteoclasts and bone resorption (Tani-Ishii et al., 1999; Zou et al., 2001), and promotes myoblasts differentiation through an autocrine process (Li and Schwartz, 2001). TNF-α activates and promotes astrocyte proliferation through a paracrine process (Rodgers et al., 2020).

### Concentration-Dependent Effects of TNF-α

Low concentrations of TNF-α tend to promote cell proliferation, whereas high concentrations tend to inhibit cell proliferation and even induce the apoptosis of neural stem cells/progenitor cells in the subventricular zone of neonatal mice, and of osteoclast progenitor cells and intestinal stem cells in fetal mice (Figure 2C; Van Der Pluijm et al., 1991; Bernardino et al., 2008; Schreurs et al., 2019). The biological effects of TNF-α are enhanced in a concentration-dependent manner. For example, higher concentrations of TNF-α inhibit the proliferation of ESCs in mice (Wuu et al., 1998), promote NPC proliferation in the human fetal cortex (Peng et al., 2008), induce the apoptosis of dopaminergic neurons in embryonic mice and chondrocytes in chicken embryos (Aizawa et al., 2001; McGuire et al., 2001; Doherty, 2007), inhibit osteogenic differentiation of BMSCs in rats (Gilbert et al., 2000, 2002; Fang et al., 2019), and promote lung branching morphogenesis and expression surfactant proteins in embryonic mice (Jaskoll et al., 1994).

### Crosstalk Between TNF-α and Other Signaling Pathways

**FIGURE 3:**
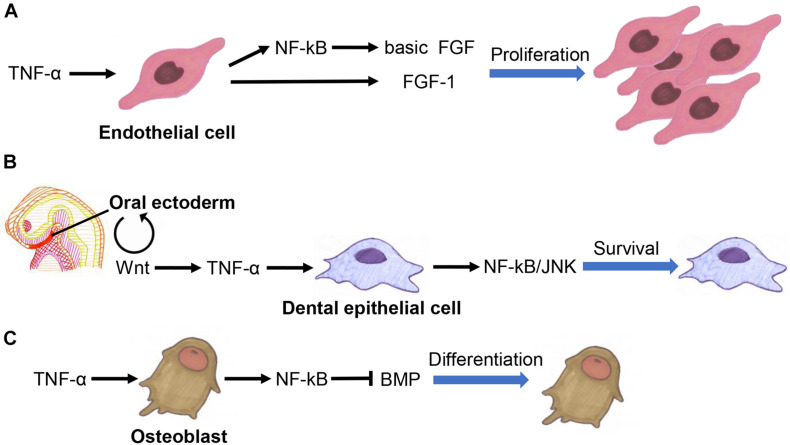
Crosstalk between TNF-α fibroblast growth factor (FGF) family **(A)**, Wnt family **(B)**, and TGF-β superfamily members **(C)** in the regulation of organogenesis.

Tumor necrosis factor alpha interacts extensively with fibroblast growth factor (FGF) family, Wnt family, and TGF-β superfamily members to co-regulate developmental processes. It promotes angiogenesis by inducing basic FGF and FGF-1 expression in endothelial cells (Figure 3A; Maier et al., 1996; Yoshida et al., 1997).

Tumor necrosis factor alpha inhibition promotes the functional recovery of nerves by activating the Wnt3a signaling pathway in BMSCs (Peng et al., 2017). TNF-α inhibits adipogenesis by activating the Wnt signaling pathway in pre-adipocytes, suggesting that it is involved in determining the fate of adipocytes (Qadir et al., 2011). TNF-α also suppresses bone formation by inhibiting the Wnt signaling in osteoblasts (Jaskoll et al., 1994; Qin et al., 2015; Chen et al., 2020; Li et al., 2020). The Wnt signaling pathway is involved in the activation of TNF-α signaling to ensure the survival of dental epithelial cells in early tooth development (Figure 3B; Laurikkala et al., 2001).

Tumor necrosis factor alpha regulates the differentiation of osteoblasts by affecting the bone morphogenetic proteins (BMP) signaling pathway (Figure 3C; Singhatanadgit et al., 2006; Mukai et al., 2007; Yamazaki et al., 2009; Matsumoto et al., 2010). It also promotes tooth development by upregulating the expression of BMP-2 and BMP-3 in dental follicles (Yao et al., 2010). Besides, BMP signaling can promote bone development by inhibiting the TNF-α-mediated apoptosis of osteoblasts (Chen et al., 2001). TNF-α induces activin A expression in BMSCs, eosinophils, lymphatic endothelial cells, and amniotic cells (Takahashi et al., 1992; Abe et al., 2013; Kelly et al., 2016; Yoshimatsu et al., 2020).

Tumor necrosis factor alpha induces the expression of hepatocyte growth factor in human bone marrow- or adipose-derived progenitor cells and in MSCs to promote tissue growth and repair (Wang et al., 2006; Zhang et al., 2010). TNF-α has also been demonstrated to promote angiogenesis by inducing the expression of ephrin A1 and erythropoietin in endothelial cells (Cheng and Chen, 2001; Wang et al., 2011). Erythropoietin can promote the proliferation and inhibit the differentiation of erythroid cells and hematopoietic stem cells by inducing the biosynthesis and secretion of TNF-α (Jacobs-Helber et al., 2003; Chen et al., 2004). Notch-activated TNF-α signaling in endothelial cells helps to prevent heart valve thickening by promoting VIC apoptosis (Wang et al., 2017).

### Inflammatory Response Triggers Abnormal Organogenesis by Activating the TNF-α

The cytotoxic effects of TNF-α during inflammation might lead to abnormal organogenesis. Previous studies have confirmed that TNF-α exerts neurotoxic effects *in vivo* and *in vitro* and negatively affects brain development *in vivo* (Chao and Hu, 1994; Peng et al., 2008; Seleme et al., 2017). Microglia activated during inflammation can inhibit the axon growth of neurons and induce neuronal apoptosis *via* TNF-α in neonatal rats (Bogdan et al., 1997; Cacci et al., 2005; Nimmervoll et al., 2013; Nolan et al., 2014; Cheng et al., 2016). TNF-α is involved in the onset of hydrocephalus, and its expression in astrocytes is associated with the severity of hydrocephalus in animal models (Jiménez et al., 2014). The induction of TNF-α expression in embryonic mice by cyclophosphamide (a teratogen) can lead to craniofacial malformations (Ivnitsky et al., 1998). Moreover, the finding that TNF-α inhibits neuronal dendritic growth in the cortex of embryonic mice might indicate increased risk of mental illness in humans (Gilmore et al., 2004; Babri et al., 2014). A high-fat diet in female rats can lead to the elevation of hepatic TNF-α to a level that can cause liver damage in their newborn infants (Kačarević et al., 2017). TNF-α promotes the maturation of pancreatic dendritic cells and activates pancreatic T cells in neonatal mice, causing damage to islet β cells and triggering the onset of type I diabetes (Lee et al., 2005). TNF-α might cause metatarsal growth disorder in fetal rats, suggesting that chronic inflammatory diseases can cause developmental disorders of bone in children by upregulating TNF-α expression (Mårtensson et al., 2004). Elevated hepatic and placental levels of TNF-α in female mice due to intrauterine infections might lead to delayed fetal bone development (Xu et al., 2006). TNF-α can increase the methylation levels of myoD CpG island in proliferating myoblasts, resulting in a reduced number of skeletal muscle cells (Sharples et al., 2016). TNF-α causes damage to the intestinal mucosa of neonatal rats by triggering the death of intestinal epithelial cells, and the subsequent onset of necrotizing enterocolitis (Halpern et al., 2006; Tayman et al., 2016; Schreurs et al., 2019). Elevated TNF-α in amniotic fluid can lead to the apoptosis of alveolar epithelial cells, localized atelectasis, alveolar inflammation, and premature birth (Sadowsky et al., 2006). All considered, abnormal factors such as inflammation in developmental processes might increase localized levels of TNF-α, and exerts cytotoxic effects that can disrupt organogenesis and trigger the onset of associated diseases.

## Discussion

This review summarized the progress in understanding the effects of TNF-α on organogenesis. TNF-α is a multifunctional cytokine that regulates important biological processes in organogenesis, such as the proliferation, differentiation, and apoptosis of neurons, osteoblasts, endothelial cells, hematopoietic progenitor cells, intestinal epithelial cells, and airway epithelial cells. TNF-α mainly inhibits the self-renewal of ESCs and promotes their migration, and inhibits the differentiation of progenitor cells and promotes their proliferation and survival. During organogenesis, TNF-α mainly acts on its secreting cells (autocrine signaling) or surrounding cells (paracrine signaling). TNF-α tends to promote cell proliferation at low concentrations and inhibit cell proliferation and induce apoptosis at high concentrations. TNF-α interacts extensively with the FGF, Wnt, and TGF-β signaling pathways to co-regulate organogenesis. It is also an important inflammatory factor that regulates the inflammatory response and exerts cytotoxic effects. The overwhelming cytotoxic effect of TNF-α during organogenesis due to inflammation interferes with normal developmental processes and can trigger disease onset. Despite considerable knowledge about TNF-α and its functions in organogenesis, several questions remain. For example, how TNF-α prevents the activation of undesirable immune responses during developmental processes remains obscure. Low concentrations of TNF-α promote the development of intestinal epithelium without triggering inflammatory response (Schreurs et al., 2019), but whether a more fine-tuned regulatory mechanism exists remains unclear. Furthermore, the regulatory mechanism underlying various effects of TNF-α (such as promoting proliferation, differentiation, and apoptosis) on the same type of cells remains unclear. Details of the regulatory mechanisms of TNF-α during organogenesis requires further investigation.

## Author Contributions

KY was in charge of acquisition of reference articles and critical revision of the manuscript for important intellectual content. HG and ZY were in charge of critical revision of the manuscript for important intellectual content. XX was in charge of drafting of the manuscript and supervision. All authors contributed to the article and approved the submitted version.

## Conflict of Interest

The authors declare that the research was conducted in the absence of any commercial or financial relationships that could be construed as a potential conflict of interest.

## Publisher’s Note

All claims expressed in this article are solely those of the authors and do not necessarily represent those of their affiliated organizations, or those of the publisher, the editors and the reviewers. Any product that may be evaluated in this article, or claim that may be made by its manufacturer, is not guaranteed or endorsed by the publisher.

## Abbreviations

ESC: embryonic stem cell
VIC: valve interstitial cell
NPC: neural progenitor cell
BMSC: bone marrow-derived mesenchymal stem cell
BMP: bone morphogenetic protein.
